# CNV Analysis of the Correlation between Preoperative Lymph Node Metastasis and Prognosis of Early Tongue Cancer

**DOI:** 10.7150/jca.60447

**Published:** 2021-08-25

**Authors:** Xi Yang, Lu Fang, Chenping Zhang

**Affiliations:** 1 Department of Oral & MaxillofacialeHead & Neck Oncology, Ninth People's Hospital, Shanghai JiaoTong University School of Medicine, Shanghai 200011, China.; 2 Genecast Biotechnology Co., Ltd, Wuxi 214104, China.; 3 Shanghai Key Laboratory of Stomatology & Shanghai Research Institute of Stomatology, National Clinical Research Center of Stomatology, Shanghai 200011, China.

**Keywords:** Lymph node metastasis, tongue cancer, copy number variation, prognosis

## Abstract

**Objective:** To investigate the relationship between preoperative concealed lymph node metastasis (LNM) and prognosis in patients with early tongue cancer.

**Methods:** According to preoperative lymph node dissection, 41 patients with tongue cancer were divided into the LNM group (*n*=19) and the non-LNM group (*n*=22). Analysis of single nucleotide variation (SNV), tumor mutation burden (TMB), mutant allele tumor heterogeneity (MATH), aneuploidy and copy number variation (CNV) was performed to identify differentially expressed genes (DEGs) related to LNM. While KEGG analysis was conducted to reveal the CNV differentially expressed genes and main enriched pathways, the correlation between pathways and genes was analyzed by hierarchical clustering. The clinical information of LNM and data on overall survival (OS) rate were obtained from The Cancer Genome Atlas (TCGA), and survival analysis was performed based on combined LNM information.

**Results:** We observed significant correlations for the mTOR signaling pathway, Hippo signaling pathway and Wnt signaling pathway with the LNM group, while leukocyte transendothelial migration and cytokine-cytokine receptor interaction were markedly correlated with the non-LNM group. Moreover, TNFRSF10C was identified as the main DEG in the leukocyte transendothelial migration pathway. TCGA-based validation revealed that the disease-free survival (DFS) of the non-LNM group was significantly higher than that of the LNM group (*P*<0.005) when TNFRSF10C CNV was set to a log2 ratio>-0.163.

**Conclusion:** The differential expression of TNFRSF10C in leukocyte transendothelial migration, an immune-related pathway, is associated with LNM and DFS. The TNFRSF10C CNV log2 ratio could potentially serve as an indicator of good prognosis for tongue cancer patients with non-LNM after neck dissection.

## Introduction

Tongue cancer is a common malignancy of the oral and maxillofacial region with a poor prognosis; most cases are squamous cell carcinoma, but its etiology remains unclear 1. Tongue cancer accounts for 33.15% of new cases of oral cancer globally, with a mortality rate of 16.02% 2. Its poor prognosis can be mainly attributed to death caused by local and distant metastases 3. Increasing evidence has shown that cervical lymph node metastasis (LNM) is one of the most important prognostic factors for oral squamous cell carcinoma (OSCC). After LNM occurs, the disease-free survival (DFS) of patients usually drops by half. Moreover, metastasis-positive lymph nodes are normally occult or subclinical during the initial treatment of early-stage tongue SCC. The speed of occult cervical LNM varies from one location of the primary tumor to another, and occult metastasis in early oral and tongue cancer cases is very extensive 4. At present, neck lymph node dissection is the primary approach for managing occult LNM in OSCC to improve the DFS of patients with early tongue cancer and metastasis-negative lymph nodes 5.

Copy number variation (CNV) refers to deletions, insertions, duplications, and complex multisite mutations in DNA fragments ranging from 1 kb to several trillion bp in size that are widely distributed in the human genome 6. The mutation rate of CNV loci is much higher than that of single nucleotide polymorphisms (SNPs), which are important pathogenic factors of human diseases 7. Tumor necrosis factor (TNF) is a cytokine that can kill tumor cells directly while exerting an antitumor effect by activating the immune system and leukocyte transendothelial migration. TNFRSF10C is a member of the TNF receptor superfamily, and studies have shown that TNFRSF10C CNV is related to distant metastatic disease 8. To date, genomic analyses of solid human tumors through projects such as the Tumor Cancer Genome Atlas (TCGA) have been conducted, providing important information about somatic changes that drive cancer progression and patient survival 9. A gene expression profile analysis found that MFAP5 and TNNC1 may serve as potential markers for predicting the prognosis of occult cervical LNM and oral tongue cancer 10. Ken et al. conducted genome analysis on the correlation between gene CNVs and occult LNM and found that loss of NKX3-1 was significantly related to occult LNM 11. However, accurate and reliable methods for predicting occult cervical lymphatic metastasis are still lacking.

In this study, we performed gene CNV and SNV analyses of early tongue cancer patients simultaneously undergoing neck lymphatic dissection to identify important markers related to LNM. Meanwhile, CNV analysis and survival analysis of TNFRSF10C were performed to determine its prognostic value.

## Methods

### Patients

A total of 41 tongue cancer patients (28 males, 13 females) were treated at our hospital. The patients were divided into an LNM group (*n*=19) and a non-LNM group (*n*=22). The inclusion criteria were as follows: (1) patients were pathologically diagnosed as squamous cell carcinoma of the tongue, at stage T1-2N0M0; (2) 18 years of age or older, no history of smoking and no history of long-term alcohol consumption; (3) LNM appeared within one year after surgery for the LNM group, and no LNM appeared within three years after surgery for the non-LNM group. Exclusion criteria were as follows: (1) patients who had not undergone a definite diagnosis; (2) patients who had received organ transplantation or blood transfusion before or after surgery; (3) patients who had a history of progression of other malignant tumors within two years prior to surgery or within two years after surgery. Patients were staged for TNM according to the 2010 edition of the American Joint Committee on Cancer. All patients underwent neck dissection. Tumor tissues and/or blood samples of patients were collected for sequencing. This study was performed in accordance with the ethical standards and the Declaration of Helsinki and according to national and international guidelines. All patients signed a written consent form, and this study was approved by the local ethics committee. Detailed clinical information of patients was in Table 1.

### Whole exome sequencing (WES) and data analysis

DNA samples were subjected to WES in Genecast Biotechnology Co., Ltd. Library was prepared via KAPA Hyper Prep Kit (Illumina platforms) (KAPA Biosystems, Massachusetts, USA). And exon regions were captured using SeqCap EZ MedExome Target Enrichment kit (Roche, Wisconsin, USA). Sequencing was conducted via Illumina Novaseq 6000 platform using PE150 strategy. Subsequently the sequencing data was trimmed adapter and filtering out low quality bases. And then clean data was aligned to hg19 human genome reference, using BWA aligner (v0.7.12) 12. Duplicate reads were marked by Picard toolkit (v2.18.2).

### Variant calling

Variants were called using VarScan2 and all variants were annotated with ANNOVAR. Somatic mutations were defined in accordance with the following criteria: (i) located in exonic regions; (ii) mutation types involved include nonsynonymous SNV, frameshift deletion, nonframeshift deletion, frameshift insertion, nonframeshift insertion, stopgain, stoploss, and splicing; (iii) allele frequency ≤0.002 in the databases ExAC 13 and genomAD 14; (iv) allele frequency ≥0.05 in the tumor sample; (v) allele depth ≥2; and (vi) absence of strand bias: there was at least one base located in one of two sites.

Copy number variants were identified using CNVkit (v0.9.2) 15 using paired mode. The generated value of log2 of the copy numbers in each interval of the BED file was then used for the following analysis.

### TCGA data collection and analysis

Data on the Head and Neck Squamous Cell Carcinoma (TCGA, PanCancer Atlas) cohort were downloaded from the cBioPortal website, and a total of 411 cases with LNM and OS (follow-up within 10 years) data were selected. All these cases were grouped according to the median CNV value of the TNFRSF10C gene, and survival analysis was conducted by combining the grouping information of LNM and non-LNM.

### TMB, MATH and aneuploidy score

To determine TMB 16, all base substitutions and indels in the coding region of targeted genes were counted according to the criteria, including nonsynonymous, frameshift and stopgain alterations. TMB and variant allele frequencies (VAFs) were calculated as mutations per megabase and the ratio of alternate allele observations to the read depth at each position, respectively. The Mutant Allele Tumor Heterogeneity (MATH) score 17 was modified to include all somatic variants with VAF between 0.02 and 1, which were calculated as 100 × median absolute deviation/median of the VAF. Aneuploidy 18 was calculated based on arms' copy number analysised via segment module with the segmentation algorithm none in CNVkit (v0.9.2). Arms with copy number ≥3 (gain) or ≤1 (loss) were defined as “altered” and altered arms were summed as Aneuploidy score.

### KEGG pathway enrichment analysis

KEGG is a commonly used online database for pathway analysis. And R package ClusterProfile was introduced to do KEGG enrichment analysis. Fisher's exact test was used in the analysis with a *P* value<0.05 set as the threshold for statistical significance. Hierarchical clustering was performed to visualize the difference in log2ratio of copynumber and SNV mutation number scaling values between the main enriched pathways of the two sets of samples.

### Statistical analysis

Kaplan-Meier analysis was used to estimate the DFS of patients to determine the potential correlation between DFS and can by using R package survival. DFS was defined as the interval between the initial treatment and the first diagnosis of recurrence. Statistical differences in SNVs were calculated via Fisher test and the differences in log2ratio of copynumber were analyzed by using Wilcox test. Log-rank test was used for Kaplan-Meier analysis. P<0.05 indicates statistical significance. Consensus clustering was analyzed via R package ConsensusClusterPlus.

## Results

### Somatic SNVs and CNVs

**Figure 1:**
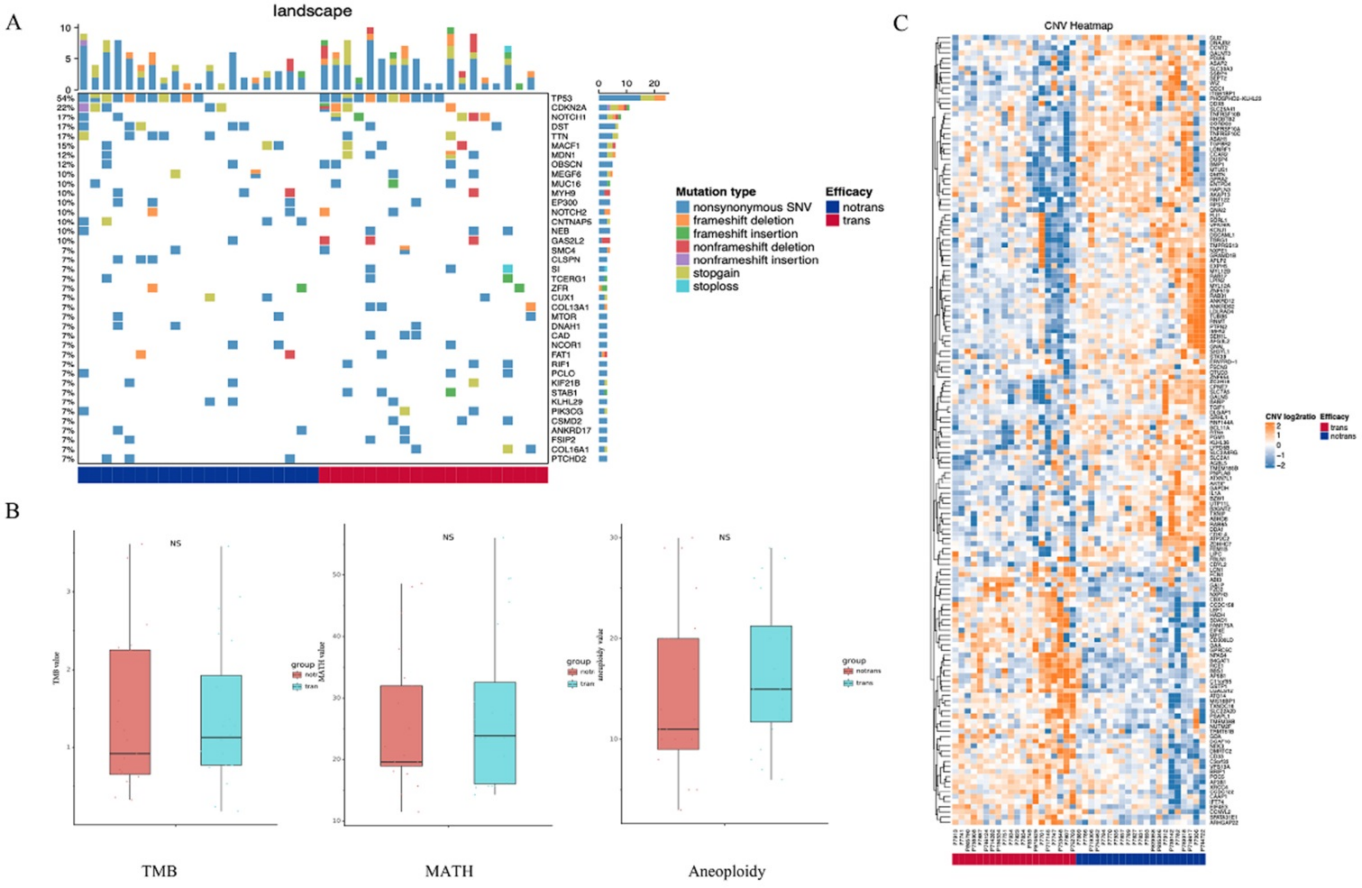
**Detection of somatic cell variation in 41 patients. (A)** The global gene mutation map of SNV testing in all patients displays gene mutations with a frequency of more than 7%. **(B)** Statistic analysis of differences in TMB, MATH and Aneuploidy genomes between the two groups of patients. **(C)** Hierarchical clustering of the DEGs based on the CNV log2ratio of the two groups of patients.

**Figure 2:**
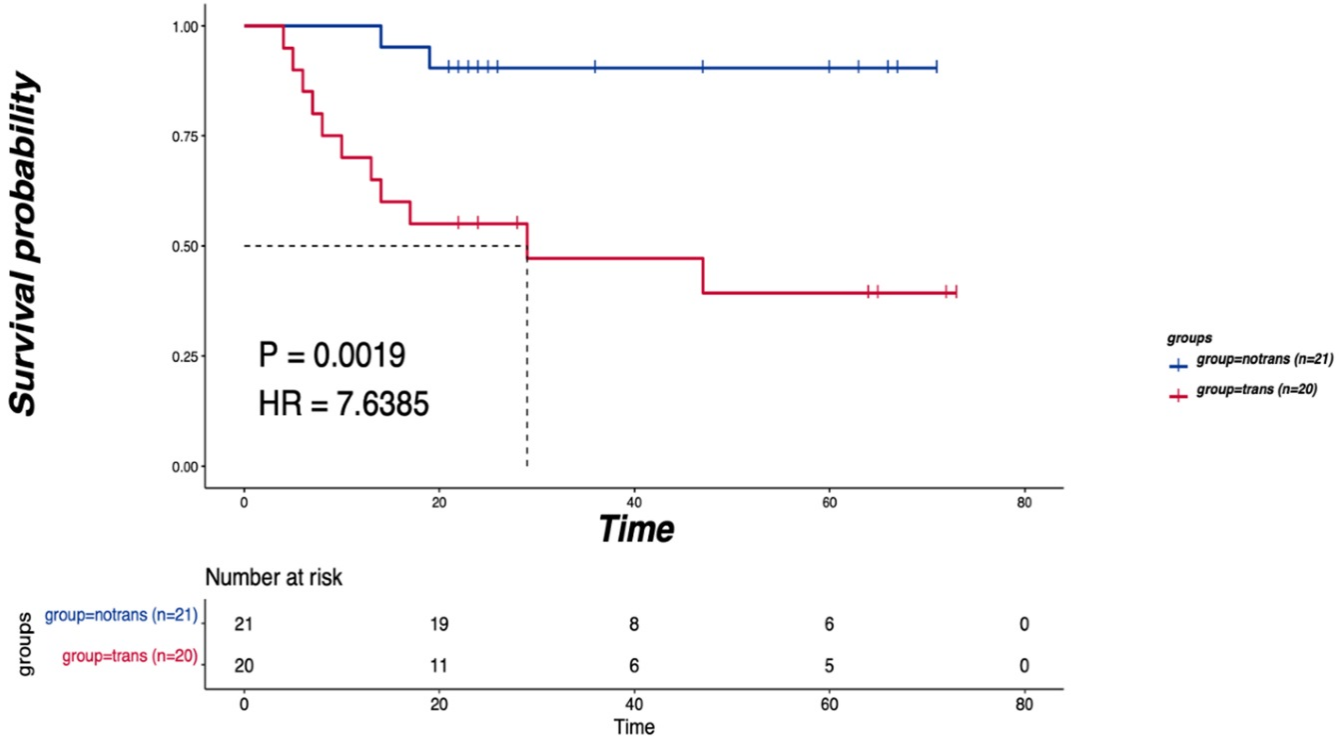
** The relationship between the LNM and DFS.** P<0.05 indicates that the difference is statistically significant.

As shown in Figure 1A, SNVs were identified in the tumor samples of 41 tongue cancer patients accoding to the method description. The global gene mutation map showed gene mutations with a population frequency of more than 7%. However, there was no significant difference in the expressed genes between the two groups (Table S1). There were no significant differences in genomic TMB, MATH or aneuploidy between the two groups (Figure 1B). The CNV difference test showed a total of 156 different genes. Among them, 51 were in the LNM group, and 105 were in the non-LNM group (Figure 1C). A significant difference in DFS was identified between the LNM and non-LNM groups (*P*=0.0019, Figure 2).

### KEGG enrichment analysis

**Figure 3:**
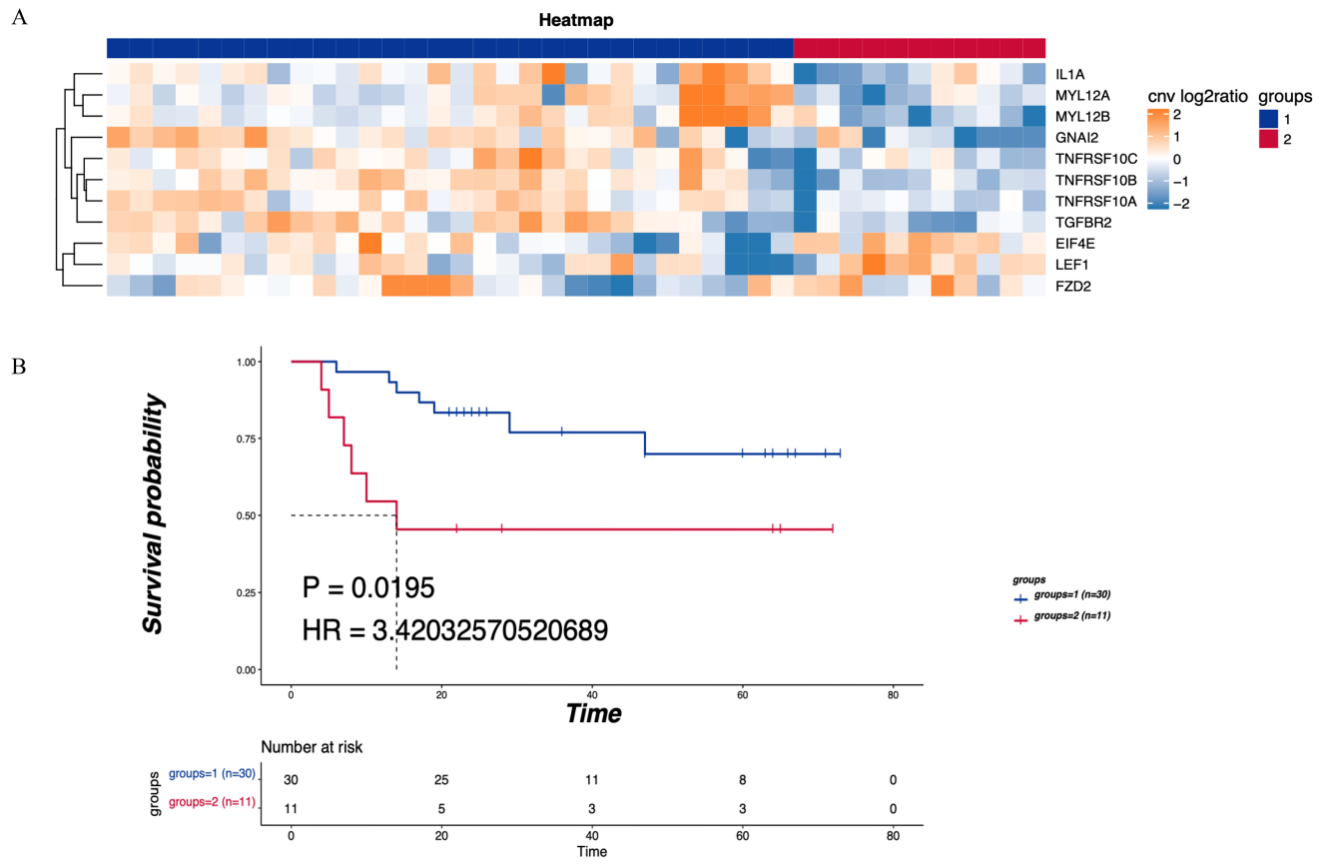
** Enriched pathways of differentially expressed CNV genes. (A)** Hierarchical clustering heatmap of 5 main enriched pathways. **(B)** Blue means CNV enhancement of immune-related pathways (n=30), red means CNV function enhancement of tumor-related pathways (n=11), and comparison of the DFS between the two groups. P<0.05 indicates that the difference is statistically significant. Group 1: CNV enhancement of immune-related pathways; Group 2: CNV enhancement of tumor-related pathways.

KEGG pathway analysis was performed on the CNV differential genome. Three main tumor-related enriched pathways were detected in the LNM group, including mTOR signaling, Hippo signaling, and Wnt signaling pathways (Table 2), while pathways related to leukocyte transendothelial migration or cytokine-cytokine receptor interaction were enriched in the non-LNM group (Table 3). As shown in Figure 3A, hierarchical clustering revealed genes related to 5 major enriched pathways, including 30 cases of CNV enhancement in immune-related pathways and 11 cases of CNV enhancement in tumor-related pathways. There was a significant difference in DFS between patients with enhanced CNV in immune-related pathways and patients with enhanced CNV in cancer-related pathways (*P* = 0.0195, Figure 3B).

### The correlation between tumor-related pathways and LNM and prognosis

**Figure 4:**
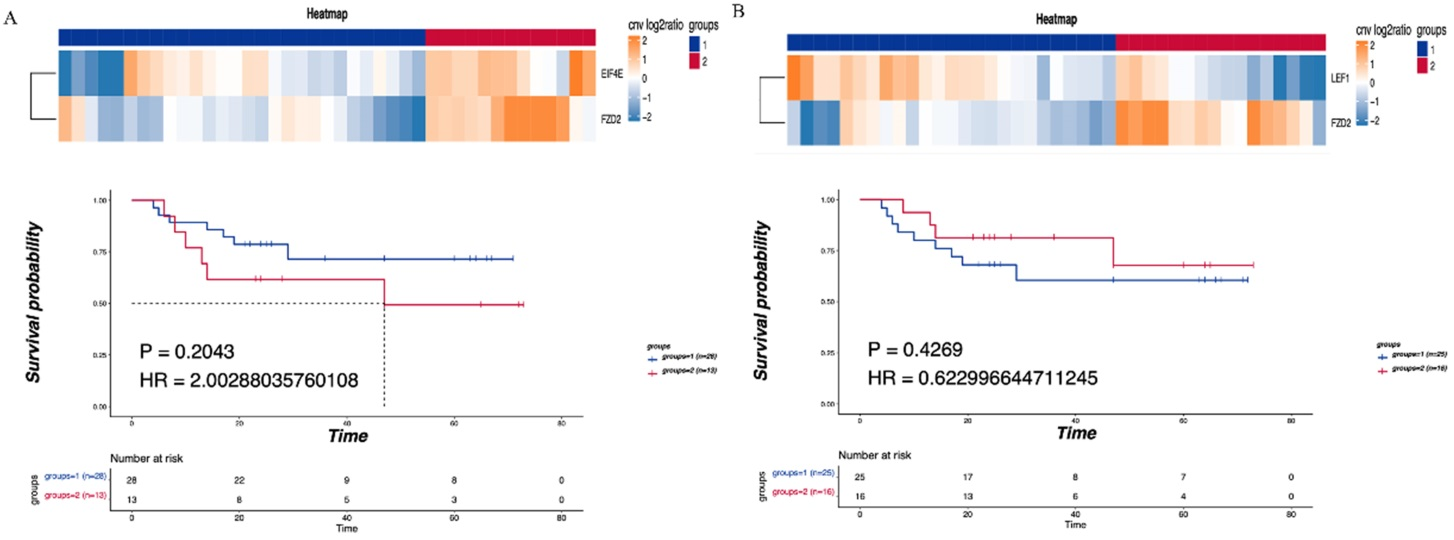
** Correlationship between tumor-related pathways and the DFS. (A)** Consensus clustering of mTOR signaling pathway, blue means CNV was not enhanced, red means CNV was enhanced, and the DFS of the two groups was compared. **(B)** Consensus clustering of Hippo/Wnt signaling pathway, blue means CNV was not enhanced, red means CNV was enhanced, and the DFS of the two groups was compared. P<0.05 indicates that the difference is statistically significant. Group 1: LNM; Group 2: non-LNM.

Consensus clustering was performed on the identified CNV tumor-related pathways to analyze the relationship between the related genes and prognosis. The mTOR signaling pathway was found to be associated with the EIF4E and FZD2 genes. No significant difference in DFS was detected between the LNM and non-LNM groups (*P*=0.2043, Figure 4A). Meanwhile, an association of the Hippo/Wnt signaling pathway with the LEF1 and FZD2 genes was observed. There was no significant difference in DFS between the LNM and non-LNM groups (*P* =0.42693, Figure 4B).

### The correlation between immune-related pathways and LNM and prognosis

**Figure 5:**
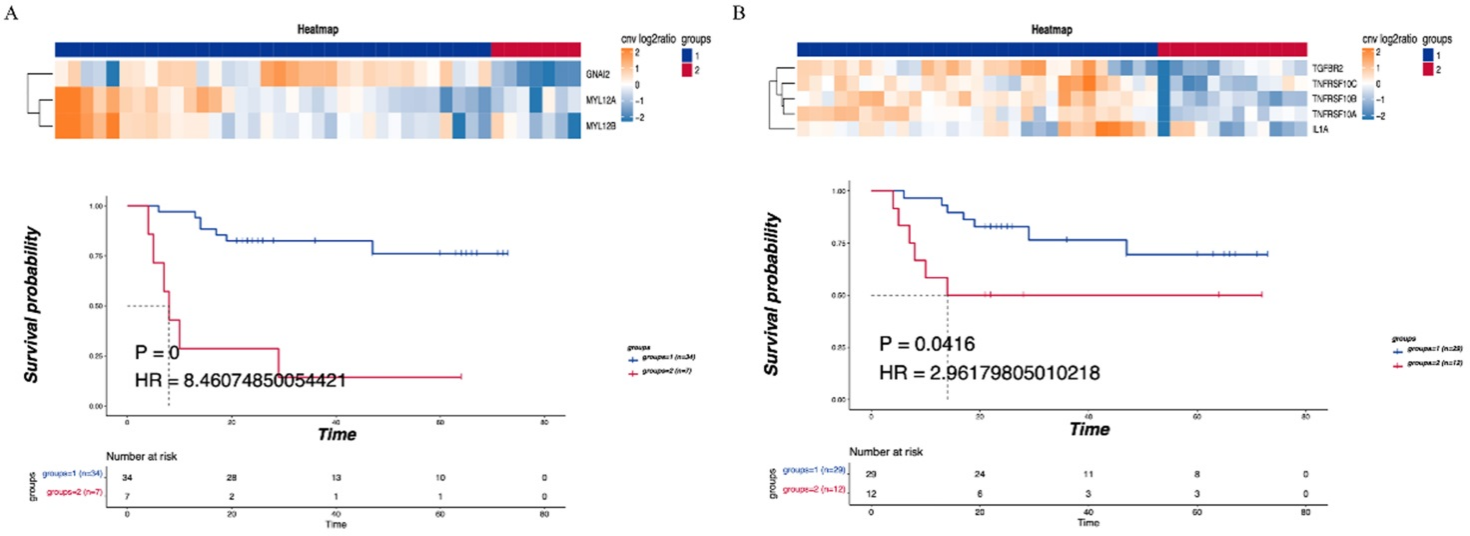
** Correlationship between immune-related pathways and the DFS. (A)** Consensus clustering of Leukocyte transendothelial migration, blue means CNV was not enhanced, red means CNV was enhanced, and the DFS of the two groups was compared. **(B)** Consensus clustering of Cytokine-cytokine receptor interaction, blue means CNV was not enhanced, red means CNV was enhanced, and the DFS of the two groups was compared. P<0.05 indicates that the difference is statistically significant. Group 1: LNM; Group 2: non-LNM.

Consensus clustering was performed on the detected CNV immune-related pathways to examine the relationship between the related genes and prognosis. Leukocyte transendothelial migration was found to be related to the GNA12, MYL12A and MYL12B genes, with a significant difference in DFS observed between the LNM and non-LNM groups (*P*=0.0000, Figure 5A). Meanwhile, cytokine-cytokine receptor interaction was shown to be related to the genes TGFBR2, TNFRSF10C, TNFRSF10B, TNFRSF10A, and 1L1A. A significant difference in DFS was evident between the LNM and non-LNM groups (*P*= 0.0416, Figure 5B).

### The correlation between TNFRSF10C CNV changes and LNM and prognosis

**Figure 6:**
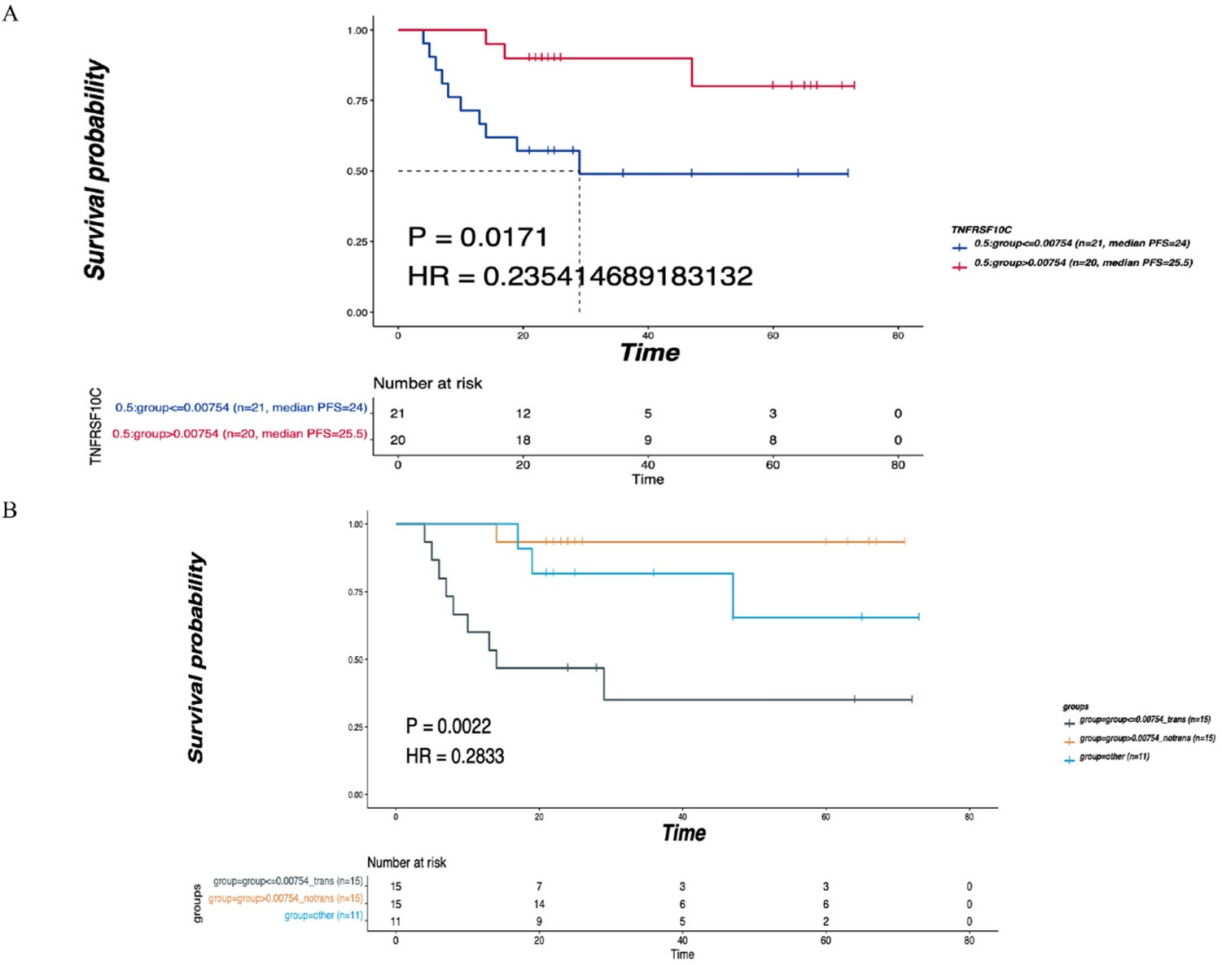
** Correlation of TNFRSF10C CNV with the LNM and DFS. (A)** According to the median value of CNV, all patients were divided into two group with log2ratio ≤ 0.00754 (n=21) and CNV log2ratio>0.00754 (n=20), and the DFS of the two groups were compared. **(B)** According to the median value of CNVs, 41 patients were divided into the LNM group (CNV log2ratio ≤ 0.00754, n=15), non-LNM group (CNV log2ratio>0.00754, n=15), other group (n=11), and the DFS of the three groups was compared. P<0.05 indicates that the difference is statistically significant. Other groups: concatenation of CNV log2ratio>0.00754_trans group and CNV log2ratio ≤ 0.00754_notrans group, as there was no significant survival difference between these two groups.

TNFRSF10C was identified as the main differentially expressed gene (DEG) in leukocyte transendothelial migration, an immune-related pathway. According to the median value of CNV, all patients were divided into two groups according to a CNV log2 ratio≤0.00754 (*n*=21) and a CNV log2 ratio>0.00754 (*n*=20). There was a statistically significant difference in DFS between the two groups (*P*=0.0171, Figure 6A). Then, 41 patients were divided into the LNM group (CNV log2 ratio≤0.00754, *n*=15), non-LNM group (CNV log2 ratio>0.00754, *n*=15), and other group (*n*=11) based on the median value of CNVs (log2 ratio=0.00754). As depicted in Figure 6B, the differences in DFS among the three groups were statistically significant (*P*=0.0022).

### Validation of TNFRSF10C in the TCGA cohort

**Figure 7:**
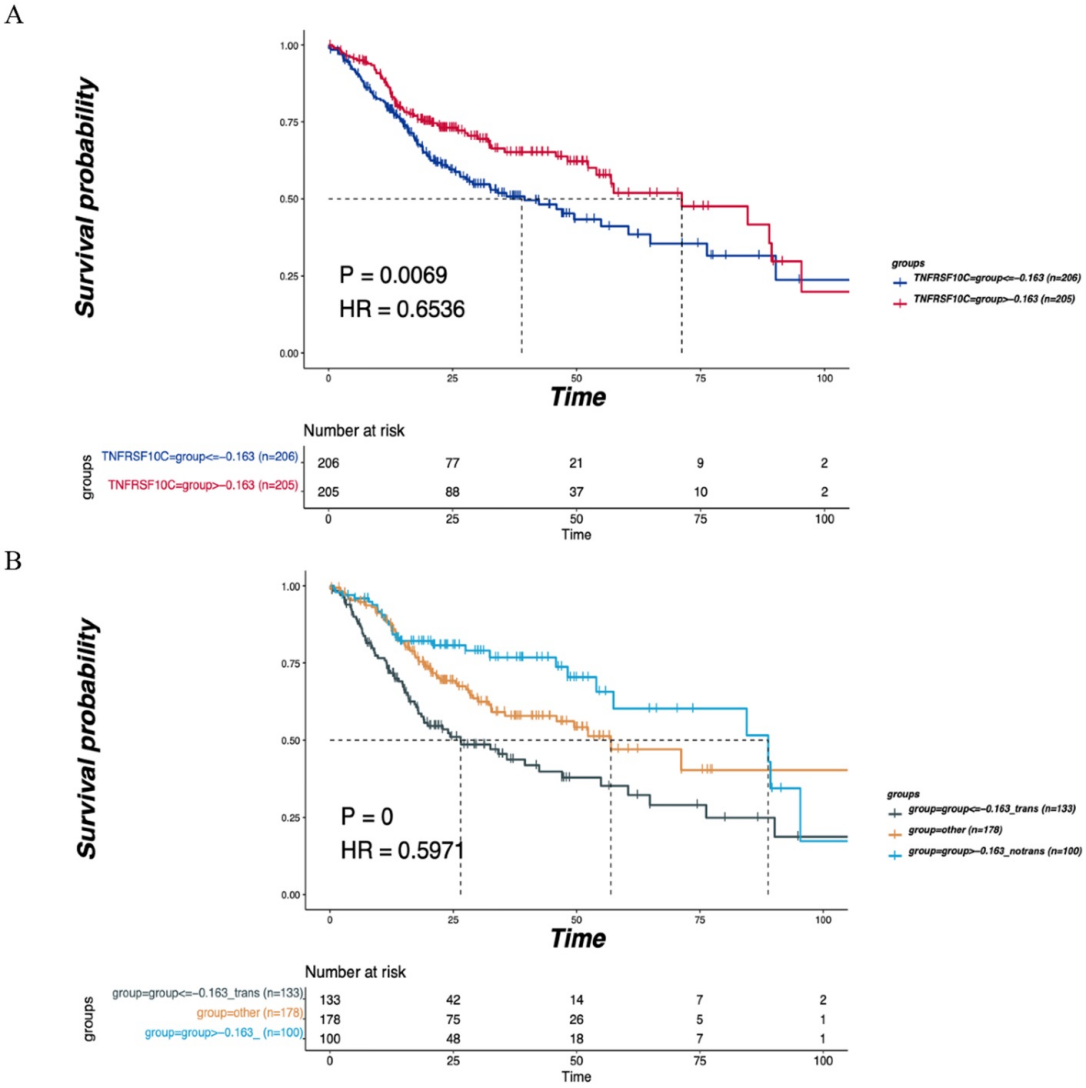
** Verification of the correlation of TNFRSF10C with the LNM and DFS. (A)** According to the median value of CNVs, all patients were divided into two group with CNV log2ration≤-0.163 (n=206) and CNV log2ration>-0.163 (n=205). The DFS of the two groups was compared. **(B)** The cases were divided into the LNM group (CNV log2ratio ≤ -0.163, n=133), non-LNM group (CNV log2ratio>-0.163, n=100) and other group (n=178) based on combined CNV median value and LNM information. The DFS of the three groups was compared. P<0.05 indicates that the difference is statistically significant. Other groups: concatenation of CNV log2ratio>-0.163_trans group and CNV log2ratio ≤ -0.163_notrans group, as there was no significant survival difference between these two groups.

A total of 411 cases with clinical information on LNM and OS were selected from the Head and Neck Squamous Cell Carcinoma (TCGA, PanCancer Atlas) cohort. These cases were grouped based on the median CNV value of the TNFRSF10C gene (CNV log2 ratio=-0.163), and survival analysis was conducted by combining LNM and non-LNM grouping information. All samples were divided into two groups based on a CNV log2 ratio≤-0.163 (*n*=206) and a CNV log2 ratio>-0.163 (*n*=205) according to the median value of CNVs. There was a statistically significant difference in DFS between the two groups (*P*=0.0069, Figure 7A). LNM information was used to further divide the samples into 3 groups: the LNM group (CNV log2 ratio≤-0.163, *n*=133), non-LNM group (CNV log2 ratio>-0.163, *n*=100), and other group (*n*=178). A significant difference in DFS was detected among the three groups (*P*=0.0000, Figure 7B).

## Discussion

CNV is a structural variation associated with multiple clinical phenotypes in the human genome. Recent studies have demonstrated that the CNV gene is significantly related to a variety of complex diseases, and CNV analysis is of great value to the treatment and prognosis of head and neck squamous cell carcinoma (HNSCC) 19, 20. In this study, 41 tongue cancer patients undergoing cervical lymphadenectomy were divided into the LNM group and non-LNM group and then subjected to an analysis of CNV differences. The analysis identified a total of 156 genes with differential CNVs. KEGG analysis revealed that leukocyte transendothelial migration and cytokine-cytokine receptor interaction, the main immune-related enriched pathways, were significantly related to LNM as well as the DFS of patients. Moreover, TNFRSF10C was identified as one of the significantly DEGs in cytokine-cytokine receptor interactions. We further used the TCGA cohort to validate CNV changes in TNFRSF10C and to analyze the relationships of CNV changes with LNM and patient prognosis.

Previous studies have suggested that leukocyte transendothelial migration and cytokine-cytokine receptor interactions are among the important pathways involved in the LNM of tumors 21. Here, we present results that are consistent with this observation. Leukocyte transendothelial migration occurs in the micron-sized spaces in the venules of the specific capillaries produced by migrating white blood cells 22 and is closely associated with the inflammatory response, multiple sclerosis and other regulatory abnormalities 23, 24. However, there is no evidence that the differentially expressed CNV genes GNA12, MYL12A, and MYL12B in leukocyte transendothelial migration are significantly correlated with occult LNM, and the underlying mechanism needs to be further studied. Zhao et al. 25 performed a pathway analysis on the gene expression profile of metastatic lymphatic samples and identified cytokine-cytokine receptor interaction as a significantly uncontrolled pathway in lymphatic metastasis. It has also been reported that dysregulated cytokine interactions are involved in the pathogenesis of cancer, as such biological processes and pathways play a key role in proliferation, angiogenesis, immune response and progression 26. CNV analysis showed that DEGs are the most abundant in the cytokine-cytokine receptor interaction pathway, and TNFRSF10C plays an important role in this pathway.

TNFRSF10C is related to the TNF superfamily human ligand-receptor interaction pathway and gene transcription pathway, and its expression is usually downregulated in cancer 27, 28. The loss of TNFRSF10C makes cells susceptible to apoptosis induced by TNF-related apoptosis-inducing ligand 29. TNFRSF10C methylation can predict the overall survival of cancer patients 30, while the expression of TNFRSF10B can serve as a good predictor for the response of cancer cells to drug treatment 31. In the present study, bioinformatics-based functional enrichment analysis revealed that TNF could serve as the central gene of the cytokine-cytokine receptor interaction pathway 32. Interestingly, we observed that according to the median value of the CNV log2 ratio (0.00754), the DFS of patients with CNV enhancement of TNFRSF10C was significantly longer than that of those without enhancement, suggesting that CNV enhancement of TNFRSF10C may prolong the DFS in patients with tongue cancer. Consistently, Zhang et al found that DFS and OS were associated with copy number changes and overactivation of the cell cycle pathway 33. TCGA-based validation (CNV log2 ratio=-0.163) showed that the DFS of the non-LNM group with a TNFRSF10C CNV log2 ratio greater than the median value was significantly improved. Similarly, Tanenbaum et al. reported that TNFRSF10C CNV was associated with distant metastatic diseases and positive lymph nodes 8. Combined with our findings showing that all non-LNM patients display a TNFRSF10C CNV log2 ratio of greater than the median value, these data provide more evidence to support the correlation between TNFRSF10C CNV and LNM.

In summary, the results of this study show that LNM is associated with leukocyte transendothelial migration and cytokine-cytokine receptor interaction, two main enriched immune-related pathways. Meanwhile, the enhancement of TNFRSF10C CNV was found to have a significant correlation with DFS in tongue cancer patients with non-LNM. Although the exact correlation between TNFRSF10C CNV and DFS must still be validated, the CNV log2 ratio>-0.163 of TNFRSF10C could potentially serve as an important marker for a good prognosis in tongue cancer patients with non-LNM after cervical lymphadenectomy.

## Supplementary Material

Supplementary table S1.

## Figures and Tables

**Table 1:** Detailed clinical information of patients

Characteristic	LNM group, n (%)	non-LNM group, n (%)
All patients	19	22
**Sex**		
Male	10 (52.63)	18 (81.82)
Female	9 (47.37)	4 (18.18)
Median age (mean ± SD)	54.21±11.70	57.00±17.05
**T stage**		
1	4 (21.05)	5 (22.73)
2	15 (78.95)	17(77.27)
**Neck pathology**		
N1	10 (52.63)	0
N2b	6 (31.58)	0
N2c	1 (5.26)	0
N3b	2 (10.53)	0
Regional recurrence	8 (42.11)	0
Radiotherapy	13 (68.42)	6 (27.27)
Chemotherapy	3 (15.79)	0
Progression-free survival (mean ± SD)	30.47±25.18	37.05±20.13
Overall survival (mean ± SD)	32.05±24.12	38.95±19.57

**Table 2:** Enrichment pathway of 51 CNV enhancement genes in LNM group

ID	Pathway	Bg Ratio	P
Hsa04142	lysosome	0.017046211	0.002303350
Hsa05225	Hepatocellular carcinoma	0.021840458	0.004646780
Hsa05217	Basal cell carcinoma	0.008256759	0.007471950
Hsa00983	Drug metabolism-other enzymes	0.010121188	0.011067590
Hsa04916	Melanogenesis	0.013317353	0.018660050
Hsa03450	Non-homologous end-joining	0.001598082	0.025290094
Hsa04150	mTOR signaling pathway	0.020109202	0.040086657
Hsa04934	Cushing syndrome	0.020375549	0.041057851
Hsa04390	Hippo signaling pathway	0.020508723	0.041546858
Hsa04310	Wnt signaling pathway	0.021041417	0.043525373

P<0.05 indicates that the difference is statistically significant.

**Table 3:** Enrichment pathway of 105 CNV-enhancing genes in the non-LNM group

ID	Pathway	Bg Ratio	P
Hsa05132	Salmonella infection	0.028099614	0.010722788
Hsa04144	Endocytosis	0.031295778	0.016480459
Hsa04061	Viral protein interaction with cytokine and cytokine receptor	0.012651485	0.022509012
Hsa05142	Chagas disease	0.013317353	0.025721494
Hsa05130	Pathogenic Escherichia coli infection	0.025302970	0.032526010
Hsa04670	Leukocyte transendothelial migration	0.014782261	0.033614506
Hsa04060	Cytokine-cytokine receptor interaction	0.038220802	0.035295159
Hsa04611	Platelet activation	0.015980823	0.040908607
Hsa00561	Glycerolipid metabolism	0.006925023	0.043332312
Hsa04142	Lysosome	0.017046211	0.048012081

P<0.05 indicates that the difference is statistically significant.

## Abbreviations

LNM: lymph node metastasis
OSCC: oral squamous cell carcinoma
DFS: disease-free survival
CNV: copy number variation
SNP: single nucleotide polymorphism
TNF: tumor necrosis factor
TCGA: tumor cancer genome atlas
WES: whole exome sequencing
VAF: variant allele frequencies
MATH: mutant allele tumor heterogeneity
HNSCC: head and neck squamous cell carcinoma
