# Supplementary material for: CNV Analysis of the Correlation between Preoperative Lymph Node Metastasis and Prognosis of Early Tongue Cancer
Source: J Cancer. 2021 Aug 25;12(20):6135–44. doi: 10.7150/jca.60447 (PMC8425219; doi:10.7150/jca.60447)
Supplement: Supplementary file 1 — Supplementary table S1. [file jcav12p6135s1.pdf]

Table S1. SNV differential genes between LNM group and non-LNM group

Gene	P
CASP9	0.10694184
RIF1	0.10694184
JADE2	0.10694184
CD209	0.10694184
COL16A1	0.10694184
CAD	0.10694184
USP17L20	0.10694184
USP17L22	0.10694184
CNTNAP5	0.10694184
COL13A1	0.10694184
USP54	0.10694184
SI	0.10694184
CSMD2	0.10694184
VPRBP	0.10694184
NISCH	0.10694184
STAB1	0.10694184
GABBR1	0.10694184
USP17L19	0.10694184
USP17L11	0.13001369
USP17L18	0.13001369
USP17L20	0.13001369
MDN1	0.18361138
USP17L13	0.18361138
AHDC1	0.23170732
PTPRF	0.23170732
ZWINT	0.23170732
CHUK	0.23170732

FAM160B1	0.23170732
ATM	0.23170732
NCOR2	0.23170732
DLL4	0.23170732
ALPK3	0.23170732
C16orf62	0.23170732

P<0.05 indicates that the difference is statistically significant.
